# Functional Characterisation and Analysis of the Soluble NKG2D Ligand Repertoire Detected in Umbilical Cord Blood Plasma

**DOI:** 10.3389/fimmu.2018.01282

**Published:** 2018-06-15

**Authors:** Steven T. Cox, Robert Danby, Diana Hernandez, Raquel Laza-Briviesca, Hayley Pearson, J. Alejandro Madrigal, Aurore Saudemont

**Affiliations:** ^1^Anthony Nolan Research Institute, Royal Free Hospital, London, United Kingdom; ^2^Cancer Institute, University College London, London, United Kingdom; ^3^Churchill Hospital, Oxford University Hospitals NHS Foundation Trust, Oxford, United Kingdom

**Keywords:** NK cell, pregnancy, tolerance, NKG2D, ligand, luciferase, MICA, ULBP1

## Abstract

We previously reported that cord blood plasma (CBP) contains significantly more soluble NKG2D ligands (sNKG2DLs), such as sMICB and sULBP1, than healthy adult plasma. Viral infection or malignant transformation upregulates expression of NKG2D ligand on affected cells, leading to NK group 2, member D (NKG2D)-mediated natural killer (NK) cell lysis. Conversely, sNKG2DL engagement of NKG2D decreases NK cell cytotoxicity leading to viral or tumour immune escape. We hypothesised that sNKG2DLs detected in CBP may represent an additional fetal–maternal tolerance mechanism. To further understand the role of sNKG2DL in pregnancy and individual contributions of the various ligand types, we carried out functional analysis using 181 CBP samples. To test the ability of CBP to suppress the function of NK cells *in vitro*, we measured expression of NKG2D, CD107a, and IFN-γ in NK cells from control donors after exposure to 181 individual CBP samples and characterised the sMICA, sMICB, and sULBP1 content of each one. Furthermore, to detect possible allelic differences between samples that may also affect function, we carried out umbilical cord blood typing for MHC class I-related chain A (MICA) and MHC class I-related chain B (MICB) coding and promoter allelic types. Strongest functional correlations related to increasing concentration of exosomal sULBP1, which was present in all CBP samples tested. In addition, common MICB alleles, such as MICB*005:02, resulted in increased concentration of sMICB. Interestingly, MICB*005:02 uniquely associated with eight different promoter types. Among promoter polymorphisms, P2 resulted in the highest expression of sMICB and P9 the least and was confirmed using *luciferase* reporter assays. Higher levels of sMICB associated with lower IFN-γ production, indicating that sMICB also suppressed NK cell function. We also examined the MICA functional dimorphism encoding methionine (met) or valine (val) at residue 129 associated with strong or weak NKG2D binding, respectively. Most sMICA associated with val/val, some with met/val but none with met/met and, counter-intuitively, the presence of sMICA in CBP increased NK cell cytotoxicity. We propose a model for fetal–maternal tolerance, whereby NK cell activity is limited by sULBP1 and sMICB in CBP. The release of 129val sMICA with weak NKG2D signalling may reduce the overall net suppressive signal and break tolerance thus allowing fetal NK cells to overcome immunological threats *in utero*.

## Introduction

Natural killer (NK) cells play an important role in innate immunity, providing a first line of defence against pathogens and early detection and elimination of transformed cells. Complex interactions between NK cell receptors and potentially aberrant ligand expression on “self” tissues or cells take place continuously *via* NK cell immunosurveillance (1). Whether or not an NK cell becomes activated leading to target cell lysis depends on the overall balance of activating and inhibitory receptor stimulation (2).

**Figure 1:**
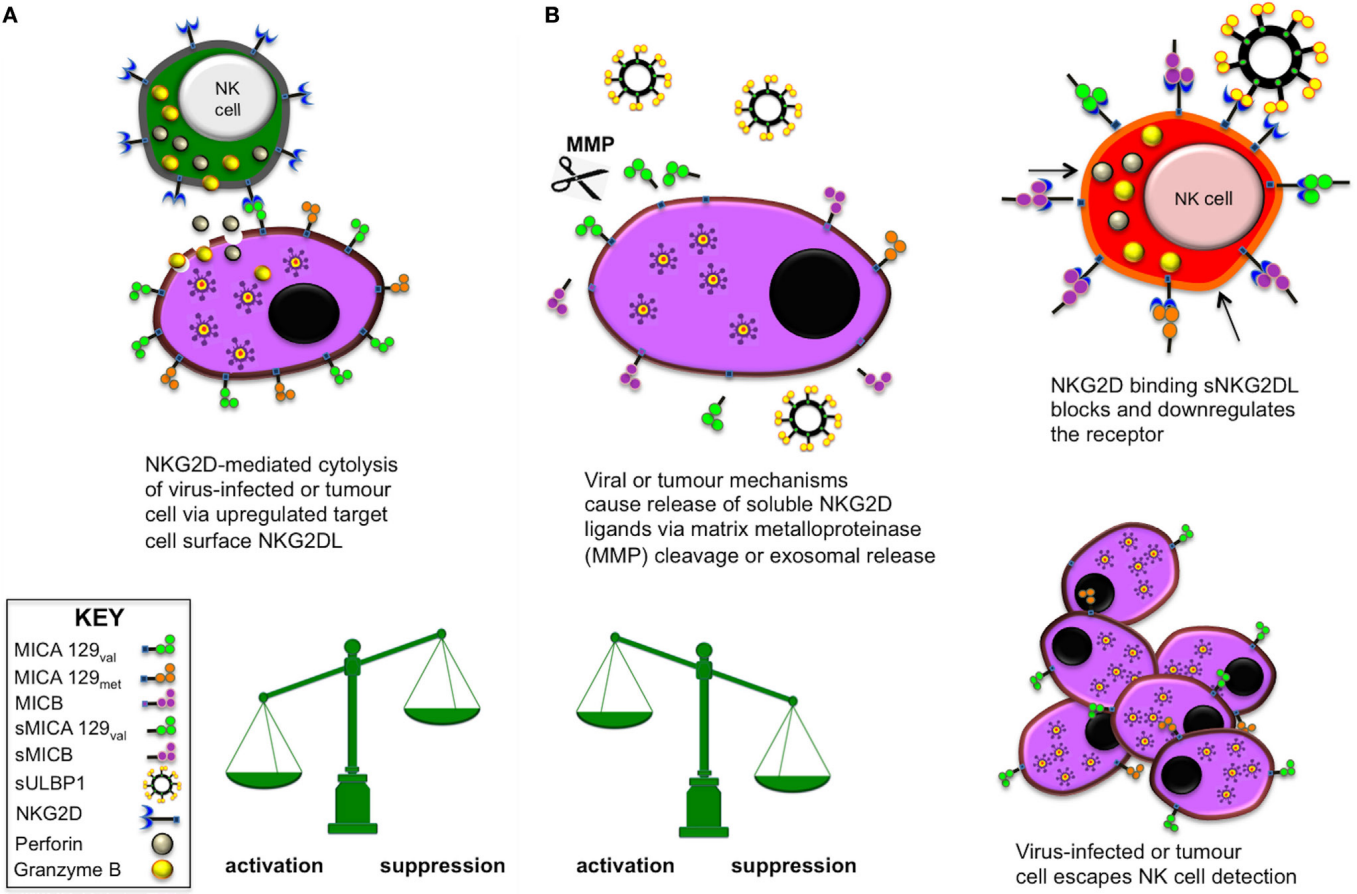
Natural killer (NK) cell activation and suppression *via* NK group 2, member D (NKG2D) engagement with membrane-bound or soluble NKG2D ligands (NKG2DLs), respectively. **(A)** When the activating receptor NKG2D on NK cells and various other lymphocytes interacts with stress-induced, upregulated NKG2DL on virus-infected or tumour cells, the target cell is eliminated by lytic actions of the effector cell. **(B)** Certain viruses and tumours are able to release soluble NKG2D ligands (sNKG2DLs) *via* MMP enzymatic cleavage or direct production of exosomal sNKG2DLs as molecular decoys. In this situation, the NKG2D activating receptor becomes blocked or is downregulated and the effector cell becomes anergic and unresponsive to further activation. This mechanism allows the tumour or virus to escape immune surveillance and proliferate.

Among the NK cell-activating receptors, the NK group 2, member D (NKG2D) receptor is perhaps the most studied but the mechanisms governing activation potential are still far from being fully understood. NKG2D interacts with ligands encoded by eight different genetic loci, including the highly polymorphic MHC class I-related chain A and B (MICA/B) and the unique long 16 binding proteins (ULBP1-6), which are also polymorphic (3–6). Apart from constitutive expression in the gut, NKG2D ligand (NKG2DL) expression is upregulated on infected and transformed cells. This enables NK cell cytotoxicity through engagement with the NKG2D activating receptor, demonstrated by studies investigating viral infection such as hepatitis B (7, 8) or cellular transformation leading to numerous types of cancer (9). Stress-induced upregulation of NKG2DL expression alone is sufficient to initiate NK cell activation and degranulation, while at the same time cytokines such as IFN-γ are released that can prime other immune cells. Viruses or tumours can avoid immune recognition by this mechanism by augmenting production of exosomal or shed soluble NKG2D ligands (sNKG2DLs) that are released into the local microenvironment. This counter-strategy successfully enables virally infected or rogue cells to escape NK cell immunosurveillance as sNKG2DL interaction with the NKG2D receptor on NK cells downregulates NKG2D expression. Thereby, the NK cell’s ability to interact with ligands *via* NKG2D is reduced but more importantly, interaction with sNKG2DLs causes NK cells to become hyporesponsive to further stimulation as shown previously by ourselves (10) and others. The opposing mechanisms of soluble and membrane-bound NKG2DLs are illustrated in Figure 1.

Soluble sNKG2DLs are essentially immunosuppressive agents targeting NK cells and other cells expressing the NKG2D receptor, such as NKT cells, γδ T cells, and CD8^+^ T cells. Such an intricate mechanism would, presumably, also have an important physiological role, such as homeostasis in immunoregulation but this has not been clearly demonstrated. These ligands, particularly MICA and MICB, are highly polymorphic but the biological significance of this diversity is still largely unknown. It is possible that MICA or MICB allelic differences between individuals may alter the potential immune response. Differences in the promoter region could alter transcriptional levels or amino acid substitutions in the molecule itself may alter protein folding or stability and influence interaction and binding with NKG2D. Thus, differences in MICA/B expression levels or their signalling through NKG2D may lead to variable NK cell function. Identification of NKG2DL polymorphisms, or alleles, that influence the immune response may enable prognosis of severity in the case of viral infection (11, 12) or cell transformation (13) or identify individuals that may benefit from immunotherapy treatment to counteract escape from NK cell immunosurveillance. In the transplantation setting, further understanding could enable better donor selection to reduce graft rejection or post-transplant complications.

We previously reported that the sNKG2DLs sMICA/B and sULBP1 are detectable in cord blood plasma (CBP) samples and responsible for decreasing adult donor NK cell cytotoxicity (10), as determined by K562 killing and chromium release assays. Therefore, it is possible that the physiological role of sNKG2DLs relates to allogeneic tolerance mechanisms in pregnancy and increasing our understanding may enable interpretation of pathologies that can arise through perturbation of NKG2DL expression *via* genetic or environmental means. This study extends our previous findings by identifying the sNKG2DLs responsible for functional inhibition of adult donor NK cell cytotoxicity. We also explore the correlation between the allelic variants and promoter polymorphisms of MICA/B genes found in umbilical cord blood (UCB), the levels of sNKG2DLs found in CBP and their potential functional consequences.

## Materials and Methods

### Subjects and Samples

Peripheral blood mononuclear cells (PBMCs) were isolated from blood of consenting healthy adult male and female volunteer donors by density-gradient centrifugation using Lympholyte^®^-H solution (Cedarlane, ON, Canada). UCB, containing acid citrate–phosphate–dextrose anticoagulant, was collected from the placenta of full-term healthy deliveries and used for DNA extraction and plasma preparation, as previously described (10). For this study, 181 different UCB units were utilised. DNA (Qiagen Blood Miniprep, Qiagen GmbH, Hilden, Germany) and plasma was isolated from each UCB unit. Four additional units were used for cord blood mononuclear cell (CBMC) isolation.

This study was carried out with the approval of the local Research Ethics Committee (reference HC71/IU). UCB units were obtained from the Anthony Nolan Cord Blood Bank with prior written consent from pregnant mothers and ethical committee approval (Research Ethics Committee reference 10/H0405/27).

### Cell Culture

For assessment of NK cell function, donor control PBMCs or CBMCs were plated in RPMI (Lonza, Slough, UK) containing 10% heat-inactivated fetal calf serum (FCS) supplemented with 1% penicillin and streptomycin (complete media) and containing human IL-2. Test cultures using 50% CBP diluted with complete media or complete media only contained 200 IU IL-2/well in 96-well plates. All cultures were incubated at 37°C with 5% CO_2_ for 48 h before PMA and ionomycin stimulation. Duplicate cultures were carried out for each experiment without PMA and ionomycin stimulation to assess levels of NKG2D and baseline CD107a. NKG2D expression on the relevant cells was determined using the unstimulated cultures and CD107a background levels in unstimulated cultures were subtracted from the values obtained for equivalent stimulated cells.

Experiments were carried out using four different, healthy PBMC donors, each tested with CBP samples from the 181 UCB units and data points represent donor means. For experiments using CBMCs, four CB units were used with seven different CBP samples. HCT116 (human colon carcinoma) used for luciferase assays were obtained from the European Collection of Cell Cultures (ECACC, Salisbury, UK). HCT116 cells were cultured in McCoy’s 5A medium (Lonza, Slough, UK) supplemented with 2 mM l-glutamine and 10% FCS at 37°C with 5% CO_2_.

### Soluble NKG2DL and IFN-γ Detection and Quantification Assays

Soluble MICA/B (DY1300/DY1599) and ULBP1 (DY1380) were detected in CBP and IFN-γ (DY285) was detected in PBMC stimulation supernatants using Duoset ELISA kits (R&D Systems, Abingdon, UK), according to the manufacturer’s instructions.

### Flow Cytometry

Briefly, cells were labelled with fluorochrome-conjugated antibodies in PBS with BSA (0.5%) for 10 min at 4°C. Antibodies (BD Biosciences, Oxford, UK) were as follows: CD3 (SK7), CD56 (B159), CD107a (HA4A3), and NKG2D (BAT221, Miltenyi Biotec, Bisley, UK). For quantitation of CD107a, cells were re-suspended in complete media containing 100 ng/ml PMA, 1 µg ionomycin and 0.1% 2-mercaptoethanol (stimulated) or complete media with 0.1% 2-mercaptoethanol (non-stimulated) for 2 h at 37°C. Fluorescence minus-one controls (where samples are stained sequentially with all antibodies except one) were used to set gates and analysis was performed using Fortessa flow cytometer (BD Biosciences, Oxford, UK) and FlowJo version 10.0.8 (Tree Star Inc., OR, USA). The gating strategy used for analysis of lymphocyte subtypes was performed as described in our previously study (10) and is shown in Figure S1 in Supplementary Material.

### Comparison of IFN-γ Production by PBMCs or Isolated NK Cells

To compare the relative amounts of IFN-γ production following PMA and ionomycin stimulation, PBMCs and NK cells from the same donor (*n* = 4) were isolated. PBMCs were prepared as described above and purified NK cells were obtained by negative selection using the NK cell isolation kit II (Miltenyi Biotec, Bergisch Gladbach, Germany) according to the manufacturer’s instructions. NK cell purity was determined by flow cytometry. PBMCs were plated at 200,000 cells/well and purified NK cells at 50,000 cells/well. Incubation with media and IL-2 was performed as described above, for 48 h. Prior to stimulation with PMA and ionomycin, cell enumeration and NK cell purity were determined for PBMC and isolated NK cell cultures. IFN-γ in the supernatants of the respective cultures was measured using ELISA as described above. PMA and ionomycin stimulation was performed for 2 h at 37°C. Results were calculated and expressed as IFN-γ (pg/ml)/10,000 NK cells.

### MICA/B Allelic and Promoter Polymorphism Analysis

Genotyping of MICA and MICB allelic and promoter type was performed on 181 DNA samples obtained from UCB as fully described previously (14).

### Generation of MICA/B Promoter Reporter Constructs

We cloned 10 MICA and 13 MICB 5′ proximal promoter fragments from sequences identified from International Histocompatibility Workshop (IHW) cell line DNA or specific populations (14, 15). MICA promoter fragments of 568 bp and 5′ MICB promoter fragments of 581 bp were amplified by polymerase chain reaction (PCR) from homozygous IHW cell line DNA or cloned using heterozygous IHW cell line or UCB DNA. The following primers were modified with restriction digest sequence tags and used for amplification (16). MICA-sense: 5′-ACTATCTACGAGCTCCGACGTCRCCACCCTCTCA-3′ (SacI underlined), MICA-antisense: 5′TGATAGATCGGTACCCAGGTGCTTCTGAGAGGCAGAGGT-3′ (KpnI underlined), MICB-sense: 5′-ACTATCTACGAGCTCCTACGTCGCCACCTTCTCAGCTG-3′, MICB-antisense: 5′-TGATAGATCGGTACCCAAGTGCTTCTGAAAGGCAGAGGC-3′. PCR conditions were 95°C for 1 min followed by 30 cycles of 95°C for 1 min, 66°C for 1 min, and 75°C for 1 min and a final extension of 75°C for 5 min. Amplified products and pGL3-Basic were treated with Thermosensitive Alkaline Phosphatase (Promega, Southampton, UK), digested with SacI and KpnI, gel extracted and ligated using T4 DNA ligase. Clones were selected after JM109 transformation and sequencing of isolated plasmids to verify integrity and promoter type. Plasmid midi preps (Promega, Southampton, UK) were then prepared, according to the manufacturer’s instructions, ready for *luciferase* experiments.

### Transient Transfection and *Luciferase* Reporter Assays

MHC class I-related chain A and MICB promoter activity was assessed with the constructs described above using dual *luciferase* reporter assays (Promega, Southampton, UK). HCT116 cells were seeded in 96-well plates at 1 × 10^5^ cells/well and grown to 50% confluence in 24 h. 100 ng of promoter pGL3-basic plasmid and 4 ng of pRL-TK (*Renilla*) was transfected per well using Lipofectamine^®^ 3000 transfection reagent (Thermo Fisher) according to the manufacturer’s recommendations. Each assay included wells transfected with pGL3-control containing SV40 promoter and enhancer (positive control) for maximal luminescence and pGL3-basic, which has no promoter and enhancer region (negative control). Activities of test promoter constructs were normalised by co-transfection with pRL-TK plasmid to correct differences in well-to-well transfection efficiency and plasmid-associated background transcription levels were determined using the negative control plasmid. A mock control (no DNA in transfection mix) was also included for subtraction of general background levels. Each test condition was assayed in triplicate for four independent experiments using three different preparations of plasmid DNA. *Luciferase* and *Renilla* luminescence was measured using a Varioskan^®^ Flash instrument (Thermo Fisher, MA, USA).

For experiments using proliferating HCT116 cells, cultures were harvested 24 h after transfection. For experiments using quiescent and heat-shocked HCT116 cells, transfection was carried out for 7 days until cells reached high confluence. Heat-shock treatment was performed by sealing plates with Parafilm and floating in a water bath for 1.5 h at 42.5°C, followed by 5.5 h recovery culture at 37°C as previously described (17).

Mock transfection luminescence was subtracted from all results. Sample and control ratios were calculated by dividing *luciferase* luminescence values by *Renilla* luminescence. Results were expressed as relative response ratios (RRR) and calculated as follows:

$$\text{RRR}=\frac{\left(\text{experimental sample ratio})-(\text{negative control ratio}\right)}{\left(\text{positive control ratio})-(\text{negative control ratio}\right)}$$

### Sequencing for MICA/B 3′ Untranslated Region (UTR) Polymorphisms

The 3′ UTR of MICA and MICB genes were sequenced to identify polymorphisms that may affect transcriptional repression by microRNAs (miRNAs). A 692-bp fragment of the MICA 3′UTR was amplified by PCR with the following primers: MICA3UFWD 5′-CCACAGGGATGCCACACAGCTC-3′ sense primer and MICA3UR 5′-CGTGCCTGGCCTGAGACT-3′ antisense primer as previously described (18). The MICB 3′UTR fragment of 1,209 bp was amplified using sense primer MICB3UF 5′-AACACCCAGTTGGGACAGGA-3′ and antisense primer MICB3UR 5′-GGAGATTGCTTTGATGCTGG-3′ as previously described (19). Amplification primers were also used as sequencing primers at standard concentration (1.6 pmol/25 µl reaction). Sequencing was carried out using a 3730XL DNA analyser (Applied Biosystems, CA, USA).

### Statistical Analysis

Results are shown as mean with SEM and were evaluated using Graphpad Prism 6 (Graphpad Software, CA, USA). Unpaired datasets were compared using the nonparametric Mann–Whitney test. Where more than two groups were compared analysis was performed using the Kruskal–Wallis test with Dunn’s *post hoc* test. Correlation and linear regression analysis significance was assessed using the nonparametric Spearman test and results are shown as *P*-values with Spearman *r* correlation value. A *P*-value ≤0.05 in two-sided tests was considered significant. Significance levels are indicated as *P ≤ 0.05, **P < 0.01, ***P < 0.001, and ****P < 0.0001, unless the exact *P*-value is given.

## Results

### NKG2D Soluble Ligands ULBP1 and MICB Present in CBP, Decrease NK Cell Functional Potential *In Vitro*

**Figure 2:**
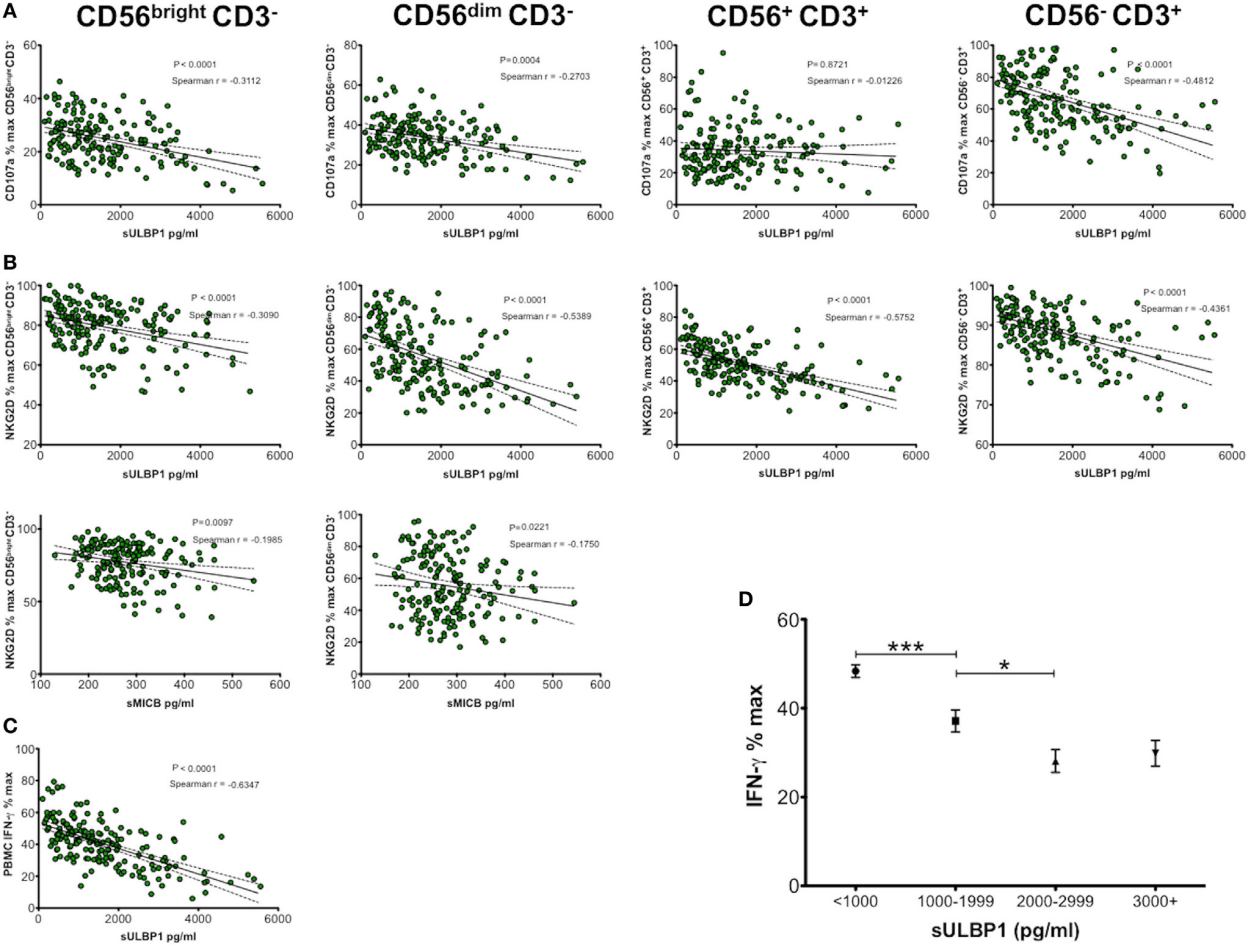
Soluble NKG2D ligand (sNKG2DL) effect on NK group 2, member D (NKG2D) and CD107a expression by CD56^bright^ CD3^−^, CD56^dim^ CD3^−^ natural killer (NK) cells, CD56^+^ CD3^+^ NKT cells, CD56^−^ CD3^+^ T cells, and IFN-γ production by peripheral blood mononuclear cells (PBMCs). **(A)** Correlation and linear regression analysis of sULBP1 concentration effect on CD107a expression. **(B)** Correlation and linear regression analysis of sULBP1 and sMICB concentration on NKG2D expression. **(C)** Correlation and linear regression analysis of sULBP1 effect on production of IFN-γ by PBMCs. PBMCs were incubated with 50% cord blood plasma (CBP) (*n* = 181) or media only for 48 h and then stimulated with PMA and ionomycin. IFN-γ in culture supernatants and sNKG2DLs in CBP was measured by ELISA and expression of CD107a and NKG2D on the different cell types was measured by flow cytometry. *P*-values for Spearman *r* correlations are indicated. **(D)** Increasing concentration of soluble ULBP1 decreases potential IFN-γ production by NK cells in a dose-dependent manner (*n* = 181). Each experiment was repeated with four different PBMC donors and data points represent donor means. Statistical analysis **(D)** was performed using Mann–Whitney test ± SEM (*P ≤ 0.05 and ***P < 0.001).

We previously demonstrated that the sNKG2DLs present in CBP decreased functional capacity of NK cells (10) but as several ligands are often present together it was not clear if more than one type was required or whether one particular ligand was responsible. To determine effects of differing sMICA/B and sULBP1 concentrations on phenotypic and functional parameters of NK cells, we cultured healthy adult donor PBMCs with media containing 181 different preparations of CBP. After 48-h culture and PMA/ionomycin stimulation, cells were analysed for expression of CD107a, NKG2D, CD56, and CD3 by flow cytometry, and the concentration of IFN-γ released into culture supernatants was measured by ELISA. The results shown in Figure 2 indicate that increasing concentration of sULBP1 in CBP significantly downregulated CD107a expression on both CD56^bright^ and CD56^dim^ NK cells and also on CD3^+^ T cells in a dose-dependent manner. However, a significant correlation was not seen with CD56^+^CD3^+^ NKT cells, although this result may be affected by low numbers of these cells. By contrast, the concentration of neither sMICA nor sMICB affected expression of CD107a whatever cell type was considered, but higher concentrations of sMICB had a moderately suppressive effect on NKG2D receptor expression in both CD56^bright^ and CD56^dim^ NK cells. Linear regression and Spearman *r* analysis showing significant sNKG2DL-mediated down-modulation of CD107a, NKG2D or IFN-γ are shown in Figures 2A–C. Increasing sULBP1 concentration also decreased NKG2D on all cell types and decreased IFN-γ levels detected in supernatants of stimulated PBMCs.

By grouping results according to ranges of sULBP1 concentrations, we found that levels below 1 ng/ml resulted in the highest levels of IFN-γ secretion of around 48% relative to complete media only controls. Increasing concentration of sULBP1 decreased detectable levels of IFN-γ in a dose-dependent manner. However, very high levels of sULBP1, exceeding 3 ng/ml, did not reduce the detectable levels of IFN-γ any further, indicating maximal functional effects when sULBP1 concentration lies between 2 and 2.9 ng/ml (Figure 2D).

### The Expression Level of CD107a and NKG2D Correlates With the Production of IFN-γ

**Figure 3:**
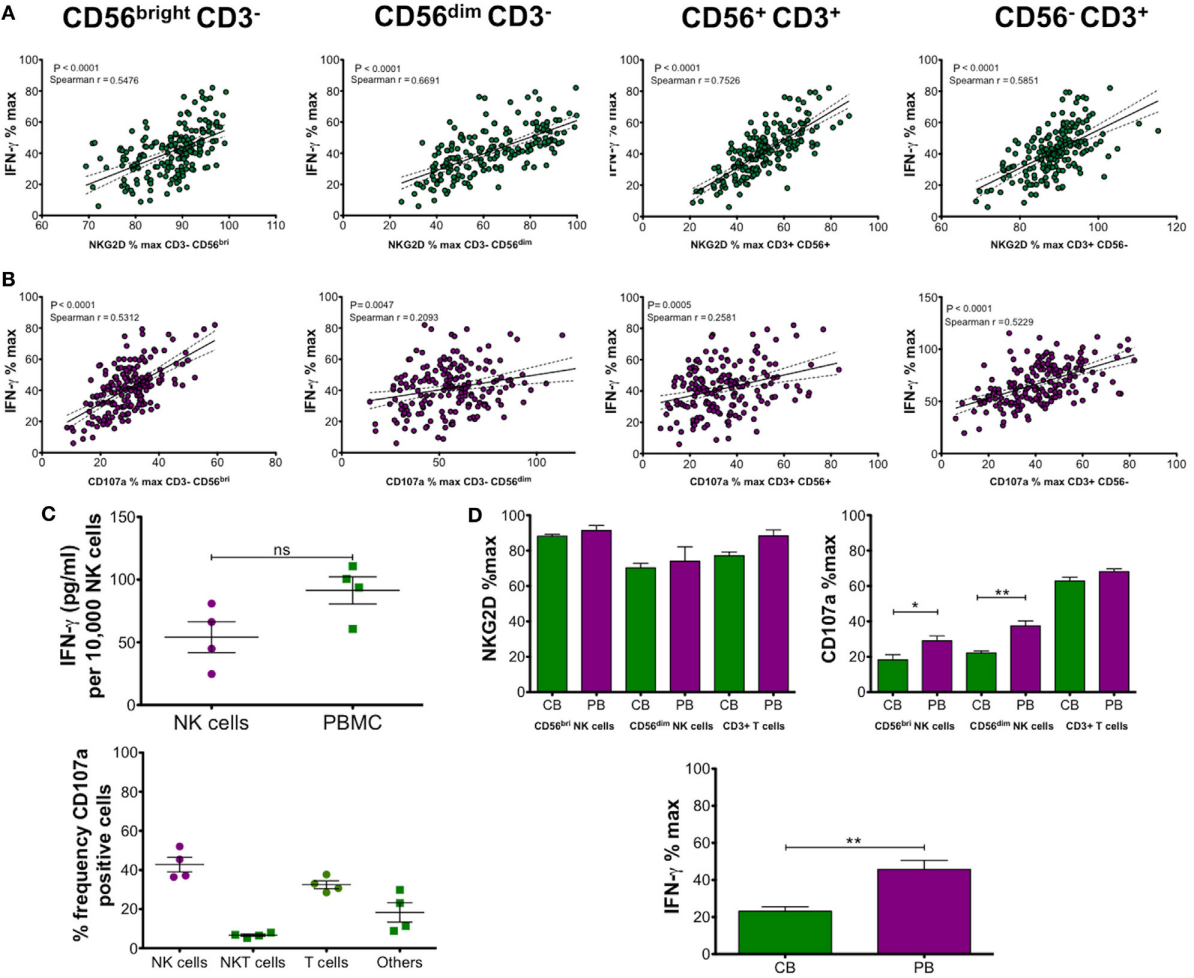
**(A,B)** Correlation and linear regression analysis of NK group 2, member D (NKG2D) and CD107a expression on CD56^bright^ CD3^−^, CD56^dim^ CD3^−^ natural killer (NK) cells, CD56^+^ CD3^+^ NKT cells, or CD56^−^ CD3^+^ T cells with IFN-γ production. Peripheral blood mononuclear cells (PBMCs) were incubated with 50% cord blood plasma (CBP) (*n* = 181) or media only for 48 h and then stimulated with PMA and ionomycin. IFN-γ in culture supernatants was measured by ELISA and expression of CD107a and NKG2D on the different cell types was measured by flow cytometry. **(C)** IFN-γ production by isolated NK cells or PBMCs was measured after CBP incubation and stimulation with PMA and ionomycin. The upper graph shows a comparison of isolated NK cells with PBMCs after calculation of IFN-γ detected by ELISA (pg/ml)/10,000 NK cells. The lower graph shows relative frequencies of CD107a-positive CD56^+^CD3^−^ NK cells, CD56^+^ CD3^+^ NKT cells, and CD56^−^ CD3^+^ T cells detected by flow cytometry. **(D)** Comparison of cord blood (CB) and adult peripheral blood (PB) derived mononuclear cells after incubation with equivalent CBP for 48 h and PMA/ionomycin stimulation (*n* = 7). NKG2D and CD107a expression relative to media only controls was measured by flow cytometry and IFN-γ detected by ELISA. Each experiment was repeated with four different PBMC donors or cord blood units and data points represent donor means. *P*-values for Spearman *r* correlations are indicated **(A,B)**. Statistical significance **(C,D)** was determined by Mann–Whitney test ± SEM (*P ≤ 0.05 and **P < 0.01).

The results shown for IFN-γ production in Figure 2 were obtained by ELISA and represent total production of IFN-γ by PBMCs following PMA and ionomycin stimulation. To determine whether there is a relationship between levels of NKG2D or CD107a and IFN-γ production, we plotted Spearman *r* correlations. The results shown in Figures 3A,B show highly significant correlations with both NKG2D and CD107a with IFN-γ. This result indicates that the suppression of NK cell function shown by reduced CD107a expression and NKG2D downregulation is directly related to the amount of IFN-γ produced by CD56^bright^ and CD56^dim^ NK cells, CD56^+^CD3^+^ NKT cells and CD3^+^ T cells.

Next, we compared the amount of IFN-γ produced by total PBMCs or isolated NK cells incubated for 48 h in RPMI containing 200 IU followed by PMA and ionomycin stimulation. Figure 3C shows the mean ± SEM concentration of IFN-γ produced by NK cells alone was 54.17 ± 12.29 pg/ml/10,000 NK cells. This compares with 91.44 ± 10.85 pg/ml/10,000 NK cells obtained by cultures using PBMCs. This result indicates that most IFN-γ is produced by NK cells and we hypothesised that the remainder was most likely the product of T cell stimulation. Since CD107a is correlated with IFN-γ production, we determined the frequency of CD107a-positive cells in media only cultures from our previous experiments to determine the relative amounts for each cell type. The results, shown in Figure 3C, show the mean ± SEM frequencies of CD107a-expressing lymphocytes were 42.7 ± 3.7 for NK cells, 6.6 ± 0.6 for NKT cells, and 32.5 ± 2.0 for T cells. The remaining double-negative gated cells accounted for 18.3 ± 4.9% CD107a-positive cells (others), which possibly represents B cells (20).

### Incubation With CBP Also Reduces Functional Capacity of CBMCs

Since this study was performed using PBMCs from healthy adult volunteers, we wanted to confirm that the effect we observed also applied to equivalent cells obtained from cord blood (CB). Using four different cord blood units, we performed the same experiments to determine percentage of maximum expression levels of NKG2D, CD107a, and IFN-γ after 48 h incubation with CBP (*n* = 7). We compared the results from CBMCs (CB) with results from PBMCs (PB) using exactly the same CBP samples. We did not include analysis of CD56^+^CD3^+^ NKT cells, as their frequency is very low, if detected at all, in CB. The results shown in Figure 3D show slightly lower levels of NKG2D in CBMCs (not significant) for NK cells and T cells. CD107a expression was significantly lower for both CD56^bright^ and CD56^dim^ NK cells in CB cells but there was no significant difference when comparing T cells. Analysis of IFN-γ also revealed significantly lower levels in CB compared with PB mononuclear cells. Overall, it can be concluded that the same effect is observed with CBMCs as with PBMCs. The significantly lower CD107a expression and IFN-γ production by CBMCs may be due to phenotypic difference between the cell types and also because CB cells are already suppressed due to the presence of sNKG2DLs in the plasma.

### MICB Allele and/or Promoter Types Affect Levels of sMICB and Functional Activity

It is possible that the highly polymorphic nature of the MICA/B genes compared with ULBP1 (which is largely conserved) could explain why we did not see strong correlations between sMICA/B levels and immunosuppressive potential, as we did with sULBP1 (Figure 2). Using DNA extracted from UCB cells, we performed MICB allele (exons 2–6) and 5′UT promoter typing as previously described (14). The aim was to establish whether allelic differences in either the promoter or the coding regions of the MICB gene influence the amount or the immunosuppressive potential of sMICB present in CBP using, where possible, UCB samples from homozygous individuals.

**Figure 4:**
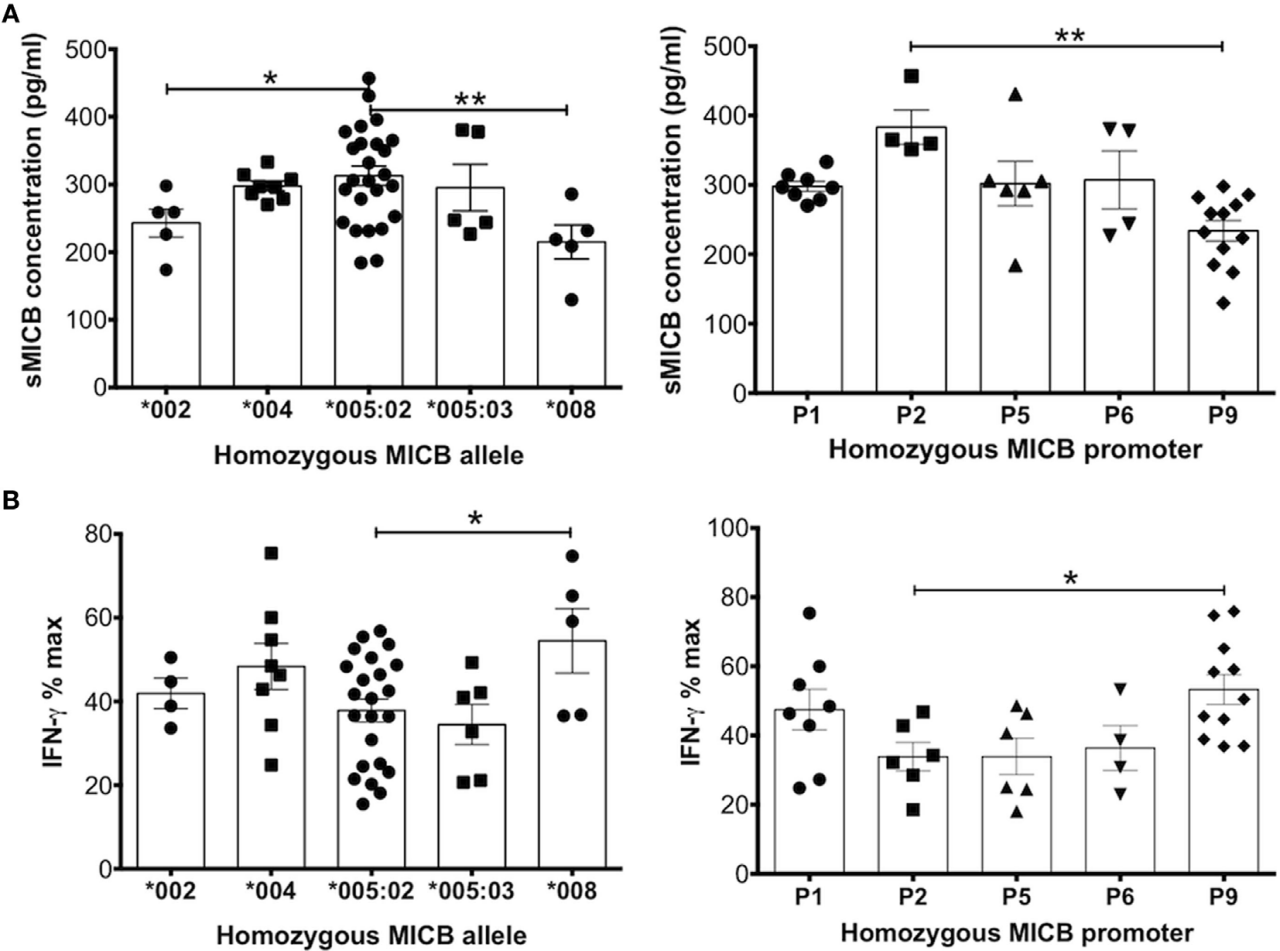
MHC class I-related chain B (MICB) allelic and promoter polymorphisms result in differential levels of soluble MICB found in cord blood plasma that influences the potential of these ligands to reduce IFN-γ production after peripheral blood mononuclear cell (PBMC) stimulation. **(A)** Levels of soluble MICB are significantly different depending on the MICB allele or the MICB proximal promoter type detected in cord blood DNA. **(B)** Similarly, polymorphic allelic differences or the proximal promoter type of MICB associate with higher or lower levels of IFN-γ. For both types of polymorphism, higher soluble MICB associates with lower IFN-γ. Data represent homozygous MICB typing obtained from a total of 181 cord blood DNA samples. Each experiment was repeated with four different PBMC donors and data points represent donor means Statistical analysis was performed using Kruskal–Wallis test with Dunn’s *post hoc* test ± SEM (*P ≤ 0.05 and **P < 0.01).

The graph in Figure 4A shows concentration of soluble MICB found in UCB from homozygous individuals for five common coding alleles of the MICB gene. The allele MICB*005:02 is highly frequent, with an allele frequency (AF) of around 60% in population studies (21, 22) and 64% in our cohort. Plasma samples from homozygote individuals with this allele show significantly higher levels of sMICB that those from individuals carrying either the *002 or *008 alleles.

We also examined the contribution of promoter types on the concentration of MICB in CBP. Promoter MICB-P2 was associated with the highest levels of sMICB and MICB-P9 with the lowest (Figure 4A). It is difficult to determine whether the higher levels of sMICB arise from structural differences in the MICB molecule or polymorphisms within the promoter region as the MICB allele*005:02 is uniquely associated with seven different MICB promoter types, including MICB-P2 (14, 15, 23), whereas all other MICB alleles are associated with only one or two promoter types (Table 1). The MICB promoter type MICB-P9 is associated with both MICB*002 and *008 and previously shown to result in reduced MICB transcription (16) owing to a two-nucleotide deletion at position -138-139 of the proximal promoter region. Since UCB typed as MICB*002/*008 with MICB-P9 promoter type shows significantly lower expression of sMICB in CBP, it is likely that promoter polymorphisms are responsible. Similarly, the promoter type MICB-P2 has a unique deletion of one nucleotide at position -126 and a G to T mutation at nucleotide position -66 and associated with significantly higher sMICB levels compared with other MICB promoter types. Therefore, to directly investigate whether promoter polymorphisms result in different levels of expression, we carried out *luciferase* assays using constructs based on most known MIC promoter polymorphisms, as described below.

**Table 1:** MICA and MICB 5′UT promoter, allele and 3′UTR allelic associations.

5′UT promoter type(s)	Allele (exons 2–6)	3′UTR type
**MICA**		
P7	001	ND
P3, P4, P7	002:01	UTR2, UTR5
P7, P8, P13	004:01	UTR1
P7	006	ND
P7, P11	007:01	UTR2
P11	007:02	ND
P1, P6, P7	008:01/04	UTR1
P7	009:01	UTR1, UTR6
P7	009:02	UTR1
P7, P9	010:01	UTR1
P4	011	UTR7
P5	012:01	UTR4
P7	015	ND
P4	016	UTR1
P3	017	UTR2
P7	018:01	UTR4
P2	019	UTR1
P4	023	ND
P7	027	UTR1
P7	033	UTR1
P10	045	UTR2
P10	059	UTR2

**MICB**		
P9	002:01	UTR1
P11	003	UTR2
P1, P3	004:01	UTR2
P1, P4	005:01	ND
P1, P2, P5, P6, P8, P9, P10, P12	005:02	UTR1
P6, P7	005:03	UTR1
P5	006	ND
P9	008	UTR1
P11	009N	UTR2
P6	013	ND
P9	014	UTR1
P10	023	ND

*ND, not determined; UTR, untranslated region; MICB, MHC class I-related chain B; MICA, MHC class I-related chain A*.

NK group 2, member D-mediated activation of NK cells occurs *via* NKG2DL upregulation on the cell surface. However, soluble NKG2DLs such as MICA and MICB block and downregulate the receptor and renders the cell anergic or hyporesponsive to activation (24, 25). In line with this concept, higher levels of sMICB in CBP should impair function of NK cells and decrease production of IFN-γ. Figure 4B shows levels of IFN-γ produced in supernatants of stimulated PBMC cultures after incubation with CBP, stratified according to homozygous MICB allele or promoter type. As expected, samples typed as MICB*005:02, having the highest sMICB levels, resulted in significantly lower IFN-γ production compared with MICB*008, which had the lowest sMICB levels and the highest levels of IFN-γ. When considering the promoter types, MICB-P9 (linked with MICB*002/*008 alleles) also showed lower levels of sMICB and therefore significantly higher IFN-γ production. Overall, it could be concluded that increased levels of sMICB decreased NK cell functional potential, as determined by lower levels of IFN-γ. In addition, it is possible that MICB promoter region polymorphism abrogated transcription factor binding, causing differences in transcription levels.

### MICA Genotype Affects Levels of sMICA and Is Associated With Increasing NK Cell Functional Capacity

**Figure 5:**
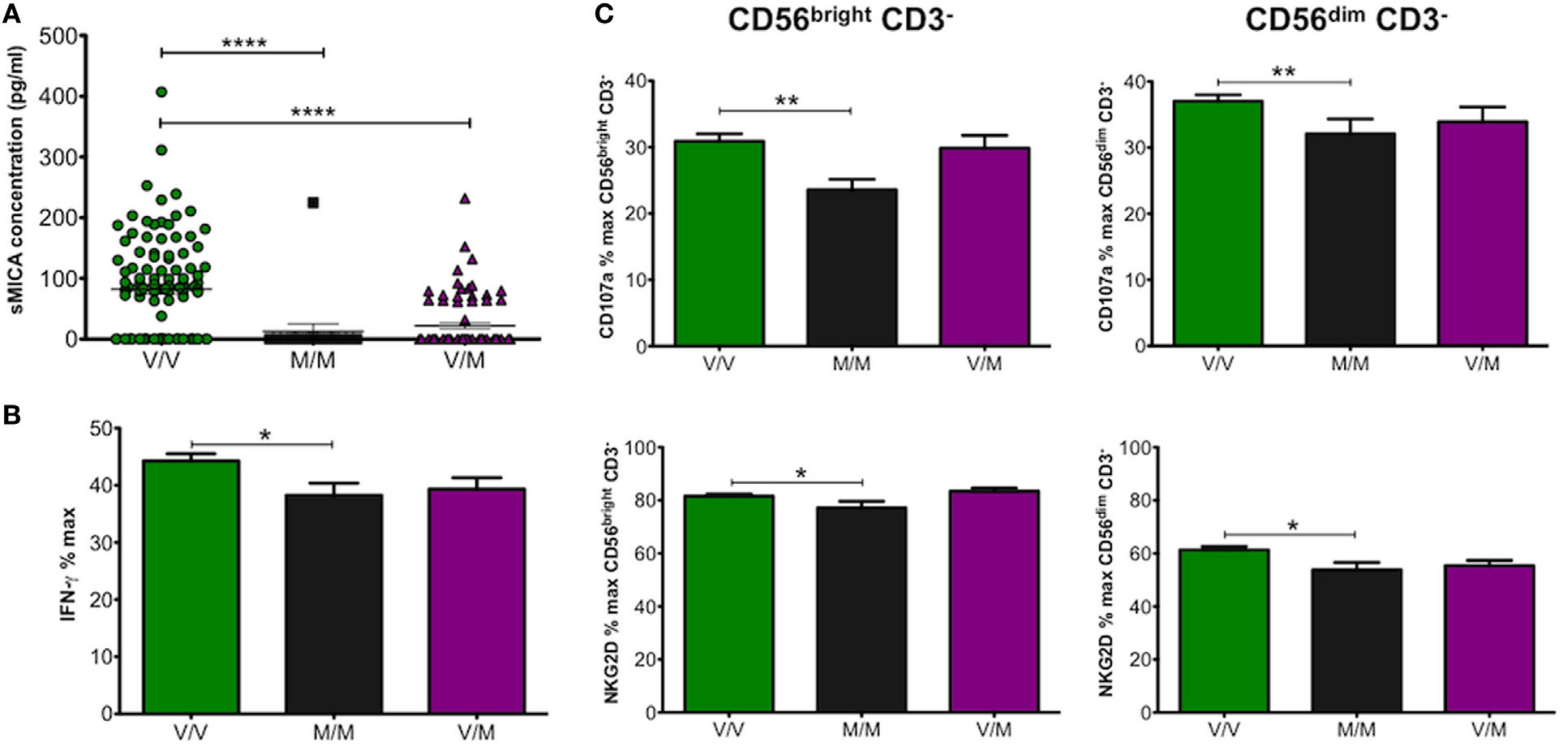
**(A)** Effect of allelic MHC class I-related chain A (MICA) differences encoding MICA-129val (V/V), MICA-129met (M/M), or heterozygotes (V/M) on concentration of sMICA (pg/ml) found in cord blood plasma (CBP) samples. Values for sMICA concentration were obtained by ELISA for each CBP sample. **(B)** Production of IFN-γ (%max) by peripheral blood mononuclear cells (PBMCs) incubated with CBP from umbilical cord blood encoding MICA allelic variants MICA-129val or -met. **(C)** CD107a and NK group 2, member D (NKG2D) levels detected on CD56^bright^ and CD56^dim^ natural killer cells. Each experiment was repeated with four different PBMC donors and data points represent donor means **(B,C)**. Statistical analysis was performed using Kruskal–Wallis test with Dunn’s *post hoc* test ± SEM **(A)** or Mann–Whitney test ± SEM **(B,C)** (*P ≤ 0.05, **P < 0.01, and ****P < 0.0001).

Unlike sMICB and sULBP1, which were detected in all CBP samples tested, sMICA was detected in only 67 of 181 samples or 37% with a mean concentration of 126.8 pg/ml. MICA allele and promoter genotyping was carried out on all UCB DNA samples (14). Most individuals are heterozygous for MICA alleles and homozygotes were mostly restricted to MICA*002 or MICA*008 in this study. Therefore, we analysed the data on the basis of methionine (met) or valine (val) amino acid presence at residue 129 of the MICA protein. This dimorphism has been shown to have functional consequences as the met protein variant has stronger binding affinity for NKG2D than val and leads to enhanced NKG2D-mediated NK cell activation (26–28). By categorising MICA genotypes as MICA-129met or MICA-129val, we found significantly higher levels of sMICA in plasma from UCB with the MICA-129val/val and 129val/met alleles, whereas almost no sMICA could be detected from MICA-129met/met UCB samples (Figure 5A). Overall, the results indicated that the presence of sMICA in CBP was restricted to UCB with genotypes encoding MICA-129val, which has weaker affinity for NKG2D. As seen with sMICB, we expected plasma samples positive for sMICA to decrease NK cell function; however, this was not the case as shown in Figures 5B,C. Cells exposed to plasma from UCB samples homozygous for MICA-129val showed significantly increased production of IFN-γ as well as higher levels of CD107a and NKG2D compared with those from MICA-129met/met or MICA-129val/met indicating enhanced NK cell function.

**Figure 6:**
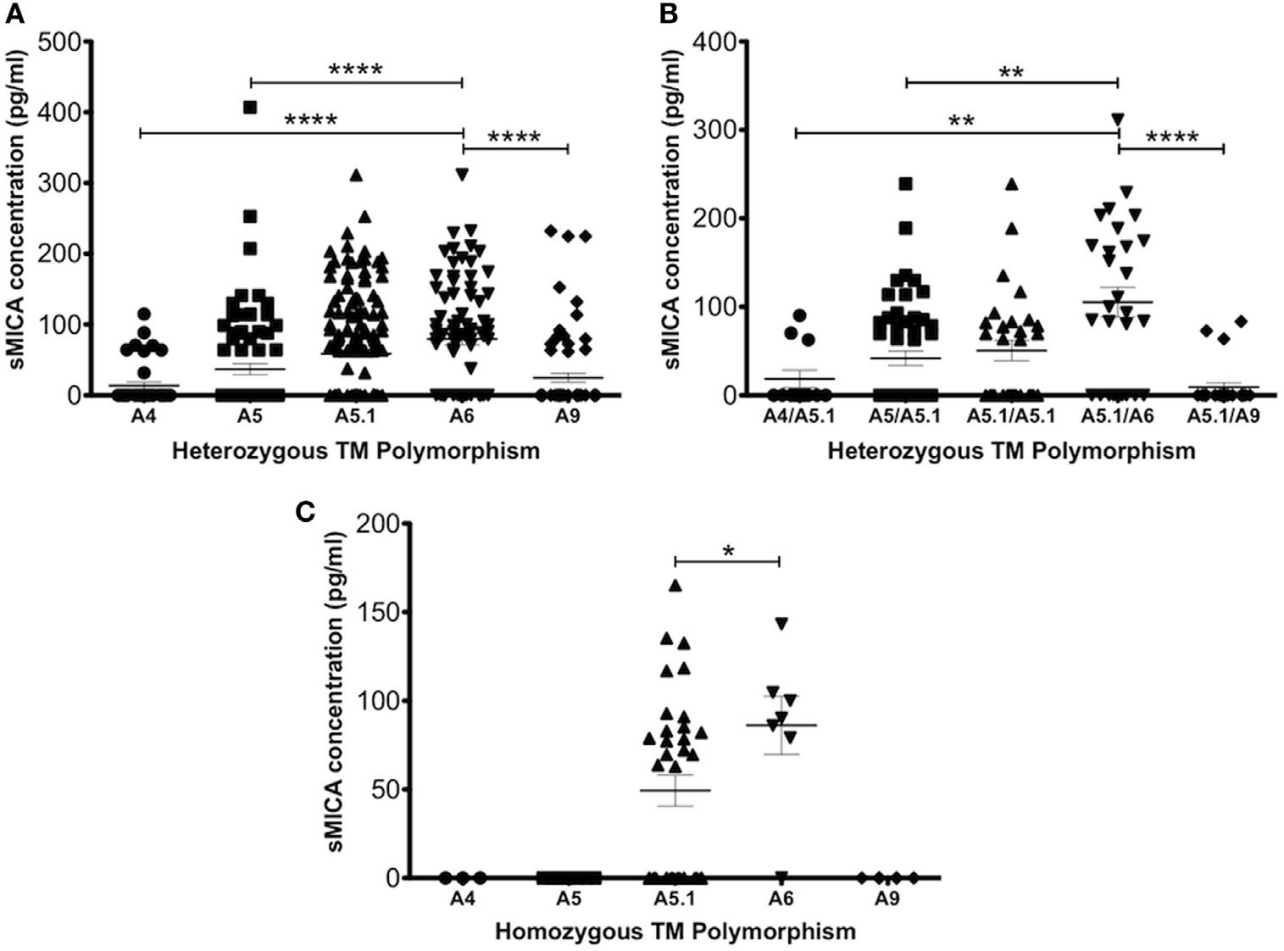
Influence of MHC class I-related chain A (MICA) transmembrane (TM) polymorphism on sMICA levels detected in umbilical cord blood (UCB) plasma samples. UCB samples were stratified according to the number of alanine residues encoded by GCT triplet (Ala) repeats in exon 5 of the MICA gene. **(A)** Heterozygous allele combinations with any other allele. **(B)** Heterozygous allele combinations with A5.1, which associates with MICA*008 with high frequency. **(C)** Homozygous TM polymorphism. Data represent MICA typing and ELISA measurement of sMICA obtained from 181 cord blood samples. Statistical analysis was performed using Kruskal–Wallis test with Dunn’s *post hoc* test ± SEM (*P ≤ 0.05, **P < 0.01, and ****P < 0.0001).

An interesting observation that may relate to shedding of soluble MICA molecules was made by stratifying samples on the basis of MICA transmembrane (TM) polymorphisms, designated A4, A5, A5.1, A6, and A9 based on the number of alanine repeats in the TM region. Type MICA-A5.1 is commonly found among MICA*008 alleles and has a nucleotide deletion leading to a premature stop codon that truncates the TM region and cytoplasmic tail. Shedding of sMICA is dependent on the disulphide isomerase Erp5 that causes a large conformational change, allowing metalloprotease cleavage (24). Figure 6A shows increasing levels of sMICA with increasing alanine repeats in heterozygotes, with the exception of MICA-A9. These results were confirmed by examining heterozygous allele combinations with the frequent MICA-A5.1 (Figure 6B). Due to insufficient homozygous samples, it was only possible to confirm that MICA-A6 associated sMICA has significantly higher concentration than MICA-A5 (Figure 6C). It is possible that the length of the TM region determines the efficiency of Erp5 and metalloprotease. For example, UCB typed as MICA-A4 had a mean concentration of 13.8 ± 4.7 pg/ml compared with 35.52 ± 9.0 pg/ml for A5 positive samples (*P* = 0.04). Maximal sMICA concentration was found with A6 (79.51 ± 8.2 pg/ml) but A9 was significantly lower having 24.6 ± 6.4 pg/ml (*P* < 0.0001), perhaps indicating A9 is too long and destabilises the complex resulting in less cleavage and less sMICA. Further investigation revealed that most MICA-129val alleles had 5 or 6 alanine repeats and most MICA-129met alleles had 4 or 9 (Table 2).

**Table 2:** MHC class I-related chain A (MICA) alleles grouped as MICA-129val or MICA-129met and their associated transmembrane polymorphisms encoding alanine repeats.

MICA-129val	MICA-129met
*004–A6	*001–A4
*006–A6	*002–A9
*008–A5.1	*007–A4
*009–A6	*011–A6
*010–A5	*012–A4
*016–A5	*015–A9
*019–A5	*017–A9
*027–A5	*018–A4
*028–A5	*020–A10
*033–A5	*026–A5
*048–A5	*029–A4
*049–A6	*030–A6
*053–A5	
*054–A5	
*056–A5	

### Transcriptional Analysis of MICA and MICB Promoters Confirms Allelic Differences Can Affect Levels of Gene Transcription

**Figure 7:**
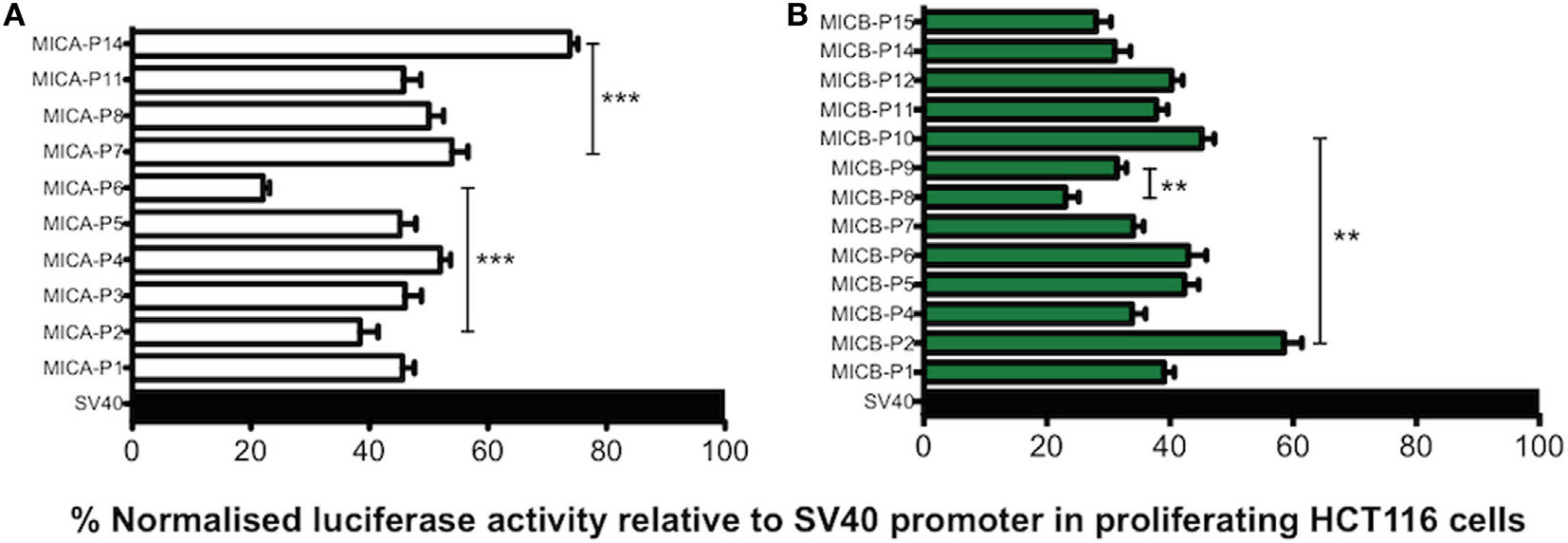
Transcriptional analysis of MHC class I-related chain A (MICA) and MHC class I-related chain B (MICB) promoter regions in proliferating HCT116 cells. Normalised percent *luciferase* activity relative to SV40 control plasmid transfection is shown. Assay conditions were performed in triplicate with three independent preparations of plasmid construct DNA and repeated for a total of four independent experiments. **(A)** MICA promoter driven *luciferase* transcription relative to SV40 promoter in proliferating HCT116 cells 24 h post-transfection. **(B)** MICB promoter driven *luciferase* transcription relative to SV40 promoter in proliferating HCT116 cells 24 h post-transfection. Statistical analysis was performed using Mann–Whitney test ± SEM (**P < 0.01 and ***P < 0.001).

To clarify how different promoter polymorphisms affect levels of transcription we performed experiments using proximal promoter polymorphic regions of MICA/B genes in *luciferase* reporter assays to determine whether sMICA or sMICB concentration in CBP corresponds to transcriptional activity. This would enable further confirmation of a likely fetal origin of sMICA/B if the transcriptional activities of the promoters correspond to sMICA/B levels found in CBP and also help identify whether any other events are involved in their shedding and release. The transcription level profiles relative to the SV40 promoter in proliferating HCT116 cells for MICA/B promoter types are shown in Figure 7. In general, slightly higher levels of around 45% RRR are seen with MICA compared with approximately 35% with MICB. Although only moderate differences are seen in transcription levels between promoter types, there are some notable exceptions for both MICA and MICB. Transcriptional activity for MICA-P6 was significantly lower than the next lowest value seen for MICA-P2 (*P* < 0.0001) and more than 50% lower RRR than other MICA promoter types. The MICA promoter that is found in association with the vast majority of MICA coding alleles is MICA-P7 and had highest RRR of all promoter types except MICA-P14. MICA-P14 was found uniquely during this study in two individual UCB samples from our cohort of 181 and is described in more detail elsewhere (29). Transcriptional activity of this promoter is high, around 75% relative to the SV40 control and higher than all other MICA promoters studied. Comparing the transcription level to MICA-P7, MICA-P14 was 20% higher (*P* < 0.0001).

MICB*005:02 promoter polymorphisms also result in variable levels of transcription. For example, MICB-P2 had the highest RRR of 58.5% compared with the next highest MICB*005:02-associated promoter MICB-P10, achieving 45% RRR (*P* = 0.002). MICB-P8 had the lowest activity of 23% and was lower than MICB-P9 with 31% (*P* = 0.004), which is known to have low expression (16). Thus, both highly frequent MICA and MICB alleles may be expressed differently depending on the polymorphisms present in the promoter region.

**Figure 8:**
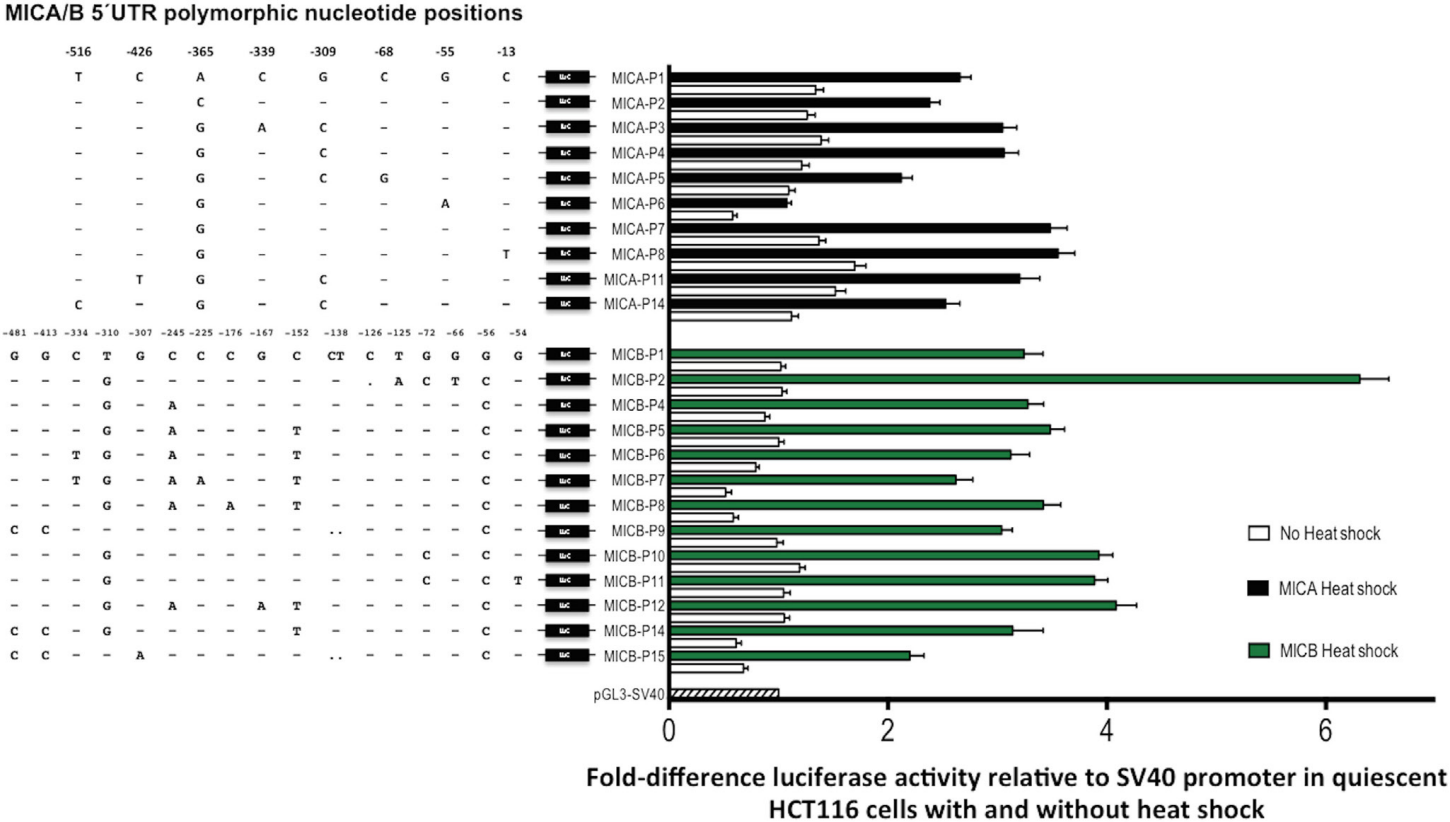
Transcriptional analysis of MHC class I-related chain A (MICA) and MHC class I-related chain B (MICB) promoter regions. 5′UTR polymorphic nucleotides associated with each type of MICA/B promoter are shown on the left and fold transcriptional activity relative to SV40 promoter is shown alongside. Quiescent HCT116 cells were grown to over-confluence for 7-day post-transfection before being heat-shock-treated (black/green bars) or isotype-treated without heat-shock (white bars). Fold-difference relative to SV40 control plasmid transfection is indicated. Assay conditions were performed in triplicate with three independent preparations of plasmid construct DNA and repeated for a total of four independent experiments. Nucleotide positions relative to ATG start codon are indicated for each polymorphic position. Abbreviations: UTR, untranslated region; LUC, *luciferase* gene.

Next, we tested for MICA/B allelic promoter transcription levels of the *luciferase* reporter gene after heat-shock treatment using a previously described method (17), whereby HCT116 cells were cultured for 7 days to over-confluence followed by heat-shock or no heat-shock treatment of these quiescent cells. Venkataraman and colleagues demonstrated that for both MICA and MICB, this core promoter region contains heat-shock elements resulting in upregulation of *luciferase* reporter gene expression and our results using allelic variants are comparable. Figure 8 shows the RRR of *luciferase* activity driven by promoter variants associated with MICA and MICB genes. Alongside these results are nucleotide alignments of polymorphic positions related to each promoter type and their relative position upstream of the ATG start codon. The unusually low transcription associated with MICA-P6 promoter remained after heat-shock and in quiescent cells. The difference between this promoter and the prevalent MICA-P7 is a nucleotide substitution of G to A at position -55 but this is not known to interfere with transcription factor-binding sites (TFBS). However, a TATA-like sequence and an activating protein 1 (AP-1)-binding site is located alongside at positions -61 to -75 as defined previously (17) and unknown transcription factors may also need to bind in this region, but this remains to be determined. MICA-P5 also has lower transcription than all other promoter types except P6 and has a C to G mutation at position -68 that interrupts a TFBS for AP-1, which may explain the lower transcriptional potential.

MHC class I-related chain B promoter types generally increased transcription under heat-shock conditions more than MICA promoters, whereas in quiescent cells *luciferase* activity was lower. MICB-P2 had the highest RRR in proliferating cells and was also much higher in heat-shocked cells at around sixfold relative to the SV40 control, compared with around threefold to fourfold for most other types. There are two consecutive nucleotide changes associated with MICB-P2 at positions -126 and -125 causing a C deletion and T to A mutation, respectively. These changes are very close to a TFBS for c-myb and the c-terminal domain of c-myb is a known transcriptional repressor (30), which may lead to overexpression of alleles driven by this promoter if c-myb binding is abrogated. The low expression indicated in proliferating cells under control of MICB-P8 was also seen in quiescent cells but heat-shocked levels of transcription were comparable to most other MICB promoter types.

Overall, these results indicate that differences in levels of transcription can occur not only between different NKG2DL genes but also between different promoter alleles of these genes, giving rise to variable expression level potential depending on an individual’s genotype. Furthermore, transcriptional regulation may vary depending on the conditions the cells are subjected to as well as the tissue origin of the affected cell.

### Differences in Levels of Transcription for Different Promoter Types Are Not Influenced by 3′UTR Polymorphisms Potentially Affecting miRNA Binding

There are currently 9 and 7 known polymorphic positions or deletions within the 3′UTR of MICA and MICB genes, respectively. Some polymorphisms occur together and have been grouped in a similar way to MICA/B promoter types, giving rise to seven types for each gene. A study of 104 unrelated, healthy Chinese Han individuals found only two types for each gene comprised the majority of this diversity (18, 19). MICA-3′UTR1 had an AF of 69.7% and MICA-3′UTR2 was 23.6%. Similarly, MICB-3′UTR1 had an AF of 79.8 and 13% for MICB-3′UTR2.

The 3′UTR harbours recognition elements for miRNAs. miRNAs are short, single-stranded noncoding molecules around 19–22 nucleotides long and either endogenous or virally encoded in origin (31). In most cases, they function to suppress gene expression during processing and *in vivo* could potentially alter results obtained using *luciferase* gene reporter assays investigating differences in promoter sequences.

To identify whether or not 3′UTR polymorphisms may affect results obtained in this study, we confirmed the results of the Chinese Han studies (18, 19) using IHW cell line DNA previously characterised for promoter and MICA/B types (14). 3′UTR polymorphism analysis was performed by PCR amplification of MICA-3′UTR (692 bp) and MICB-3′UTR (1,209 bp) followed by Sanger sequencing. The results, shown in Table 2, were in agreement with the previous studies and showed that MICA-P6 was on the same haplotype as MICA-3′UTR1, as were other promoters with higher transcriptional potential. Of particular interest, the strong promoter MICB-P2 was also on the same haplotype as MICB-3′UTR1, which was also seen with MICB-P5, P6, P9, P10, and P12. Consequently, the low expression driven by MICA-P6 or high expression driven by MICB-P2 is unlikely to be affected by polymorphisms in the 3′UTR influencing miRNA binding.

### Promoter Type-Specific Transcription Potential and Levels of sNKG2DLs Are Similar for sMICB Ligands but Not sMICA

**Figure 9:**
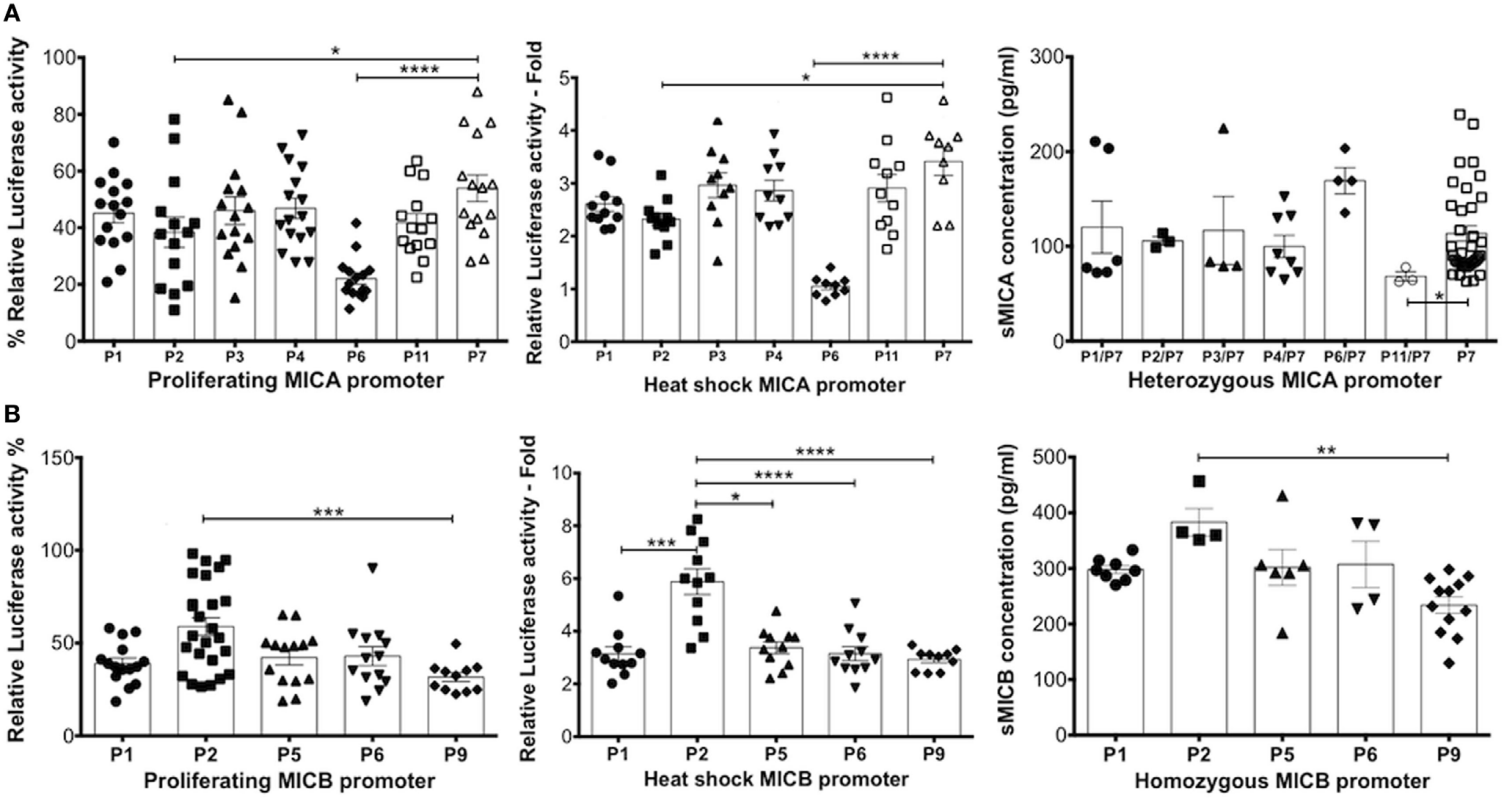
Comparison of relative *luciferase* transcription potential of MHC class I-related chain A (MICA)/MHC class I-related chain B (MICB) promoter types in proliferating and heat-shocked HCT116 cells and comparisons with levels of sMICA/B in cord blood. **(A)** Relative *luciferase* activity obtained using MICA promoter types. Owing to a lack of MICA homozygotes, heterozygous MICA promoter types were analysed in combination with the highly frequent MICA-P7 for comparison of sMICA levels with transcription levels. **(B)** Relative *luciferase* activity obtained using MICB promoter types associated with sMICB levels in homozygous MICB promoter typed cord blood. sMICA/B concentrations were obtained by ELISA for each cord blood plasma sample. Statistical analysis was performed using Kruskal–Wallis test with Dunn’s *post hoc* test ± SEM (*P ≤ 0.05, **P < 0.01, ***P < 0.001, and ****P < 0.0001).

Figure 9 shows relative *luciferase* activity detected using individual promoter types for MICA (Figure 9A) and MICB genes (Figure 9B) alongside the corresponding levels of sMICA/B detected among 181 UCB samples. Owing to a lack of homozygous promoter types for MICA, we analysed different promoter types in combination with the common MICA-P7 promoter. We found that MICA-P6 and -P2 relative *luciferase* activity was significantly lower in both proliferating and heat-shocked HCT116 cells. By contrast, levels of sMICA in MICA-P6-positive samples were significantly higher. In addition, levels of sMICA among MICA-P11 samples were significantly lower than MICA-P7 alone but transcription levels in both experimental conditions for promoter P11 were similar to most other promoter types. Furthermore, the results shown in Figure 5 show CBP with detectable sMICA associated with MICA-129val and the data presented in Figure 6 and Table 2 show a relationship between TM alanine repeats and sMICA levels. These results strongly suggest that rather than differences in transcription potential determining the amount of sMICA detected in CBP, their presence may be related to structural differences.

The analysis of sMICB ligand concentration levels from homozygous promoter typed samples and relative *luciferase* activity for individual promoter sequences showed similar profiles to both proliferating and heat-shocked HCT116 cells. In heat-shocked cells, MICB-P2 had highly significantly increased levels of *luciferase* activity compared to MICB-P1, P5, P6, and P9, with lowest levels related to MICA-P9. The profile seen relating to sMICB concentration is very similar to these transcription levels with MICB-P2 levels significantly higher and MICB-P9 significantly lower. Therefore, in contrast to MICA, it is likely that levels of sMICB are directly related to promoter strength.

## Discussion

We have previously shown that CBP contains soluble ligands for NKG2D that can influence the function of NK cells and other cells by decreasing cytotoxicity *via* their interaction with the NKG2D receptor (10). Furthermore, we demonstrated that at the time of birth, significantly higher levels of sMICA and sMICB were present in CBP than in plasma from the paired maternal peripheral blood. By contrast, higher levels of sULBP1 were found in maternal plasma than CBP, indicating a possible fetal source of sMICA/B and maternal source of sULBP1. Differences were also detected in the physical form of the ligands as sMICA/B were shown to be single soluble molecules whereas sULBP1 was found on isolated exosomes. In this study, we sought to determine which sNKG2DLs were responsible for the NK cell functional suppression. Did one type of ligand dominate in suppressive action or were other ligands needed and does polymorphism of MICA and MICB have any effect? Furthermore, can the information obtained from this analysis delineate the nature of this mechanism in terms of fetal–maternal tolerance or *in utero* immunity?

We utilised the ability of CBP containing sNKG2DLs to inhibit NK cell responses *via* NKG2D as previously demonstrated (10), choosing optimal conditions such as concentration of CBP and time of incubation to further define the biological role of the various sNKG2DLs. This time, a large cohort of 181 CBP and UCB DNA was collected and used to determine not only the type of sNKG2DLs present but also the MICA and MICB allelic and promoter types of the fetus. With this information we could then determine how differences in structure derived from allelic variants and levels of expression governed by polymorphic promoter sequences affected the functional potential of CBP to suppress the activation of NK cells, NKT cells, and T cells.

The data show that the main sNKG2DL correlating with functional suppression of NK cells and other cells expressing NKG2D is sULBP1, which is also the most abundant and, in contrast to sMICA and sMICB, monomorphic. However, stratification based on allelic types of MICB also shows a suppressive effect with some alleles having more of an effect than others. This is either a consequence of structural differences or a tendency for a particular allele to be expressed more abundantly due to promoter nucleotide polymorphisms. Indeed, the data show that differences relating to a particular allele can be explained by the variation in the promoter region. For example, sMICB*002 and *008 displayed lowest levels in CBP and both have MICB-P9 type promoter that also correlated with low relative transcriptional activity. Conversely, MICB*005:02 homozygotes had the highest concentration of sMICB in plasma and one of the promoter types associated with this allele, MICB-P2, also had high levels of expression and very high transcriptional potential. Contrary to what was expected, the two-nucleotide deletion of CT at positions -138-9 affecting MICB-P9 (and P15) did not result in substantial transcriptional reduction after heat-shock and was only slightly lower than other promoter types in proliferating cells. MICB-P15 was lower under all conditions but has an additional mutation of G to A at position -307, which may alter a TFBS. There are a number of reasons why our results may differ to those of Rodríguez-Rodero and colleagues (16). First, we used HCT116 cells, whereas Hela and CaCo-2 cells were used in the previous study, perhaps indicating that differences in cells or tissues can affect transcription. In addition, the expression plasmid used was not the same and heat-shock conditions were not tested previously. As we observed with other promoter types such as MICB-P8, consistently low expression in proliferating cells was not observed in heat-shocked cells.

Our data also showed that the higher plasma concentrations of sMICB induced the lowest levels of IFN-γ after incubation with PBMCs. However, this was not the case with sMICA allelic variants where higher levels of sMICA were associated with more activation and production of IFN-γ. Neither was there a clear relationship between the level of transcription potential and the concentration of sMICA detected in CBP. However, we did identify MICA promoters that caused high or low expression in association with the same MICA allele. In our previous study (14), we found MICA-P6 sometimes associated with MICA*008 (the most common MICA allele in most populations), rather than the more frequent MICA-P7 promoter (Table 1). Although the population frequency of this promoter type is largely unknown, one study investigating MICA allele and promoter haplotypes suggested an AF for MICA-P6 of around 3% in Chinese Han, on the same haplotype as MICA*008:01 (15). MICA-P13 and MICA-P14 were identified as a novel promoter types in our cohort of cord blood samples (29) and have the accession numbers KM358317 and KM358136, respectively. Although we cannot confirm the MICA allele haplotype association with MICA-P14, both individuals with this novel promoter were typed as MICA*008 so it is possible that this allele can be over-expressed in the presence of strong promoters such as P14. Overall, most individuals express MICA*008 moderately *via* MICA-P7 but those with the MICA-P6/*008 haplotype have very low expression and those with the MICA-P14/*008 have very high expression. Hence, MICA*008, currently defined as one allele type in terms of structure, may have diverse functional potential due to large differences in expression and have implications regarding MICA genotype and disease association studies, the immune response to tumours, infections, and transplantation in the unrelated setting.

Testing of CBP revealed that only samples typed as MICA-129val or MICA-129val/met had detectable levels of sMICA and this seemed to relate to the length of the TM region that may be influencing its ability to be shed from the as yet unconfirmed cellular source. These results may offer insights into mechanisms of *in utero* immunity that are currently very poorly understood. On one hand, immune tolerance and suppression is essential to maintain a *status quo* and avoid problems arising from allogenicity between the fetus and the mother. On the other hand, mechanisms must exist to allow immune challenge by the fetus against opportunistic pathogens in order to survive. We have derived a model based on the main findings of this study that allows sNKG2DLs to maintain tolerance under normal circumstances but also enable a break in tolerance and activation of NK cells to pursue an innate immune challenge.

**Figure 10:**
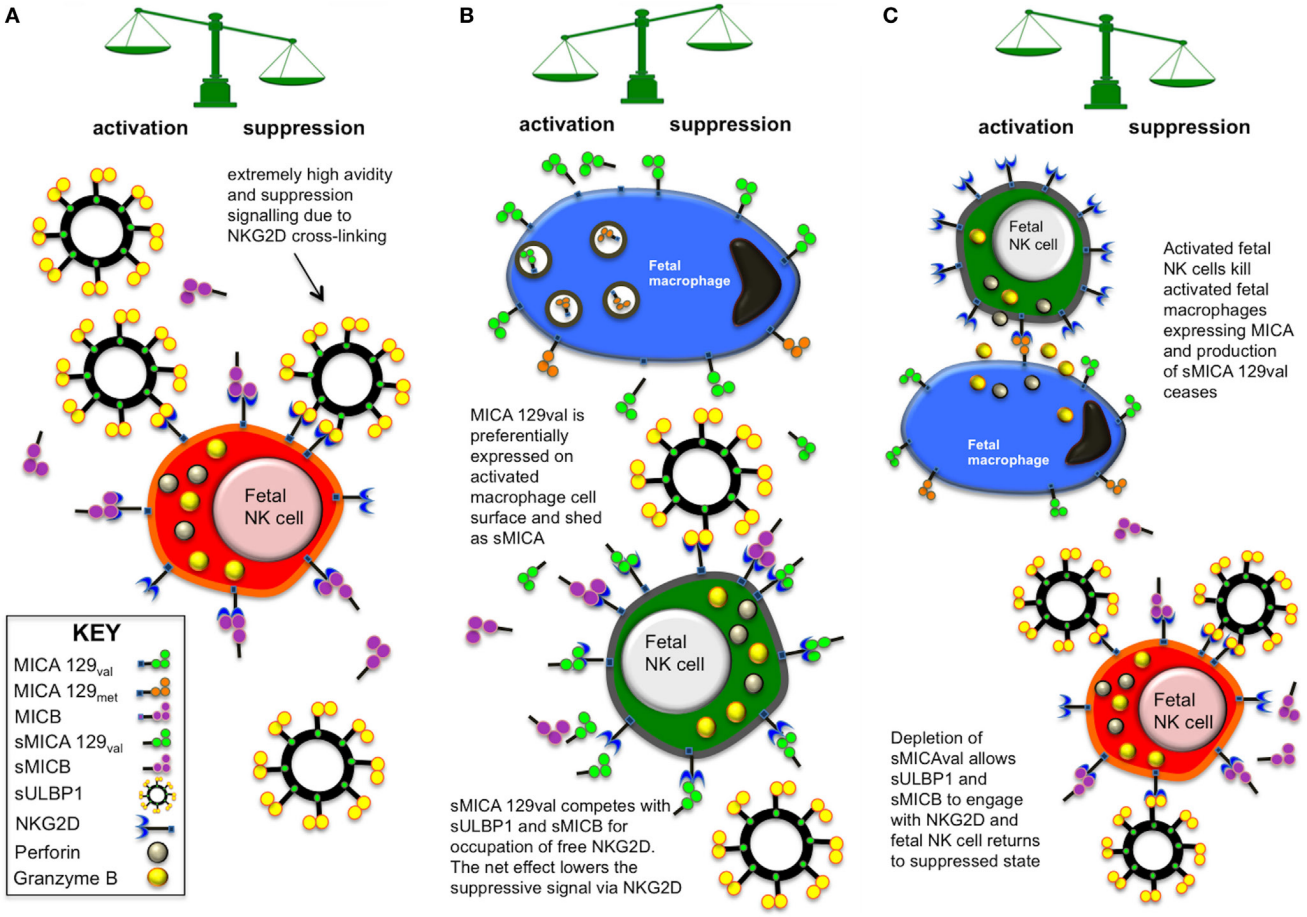
Model of *in utero* fetal immune suppression and tolerance mediated *via* soluble NKG2D ligand (NKG2DLs) interaction with fetal natural killer (NK) cells. **(A)** Under normal circumstances, an abundance of sMICB and sULBP1 binds with available NK group 2, member D (NKG2D) receptors on fetal NK cells and renders them hyporesponsive to activating stimuli at the maternal:fetal interface, promoting tolerance. In the particular, the very high concentration of high-avidity multivalent exosomal sULBP1 is a powerful suppressor of NK cells and may crosslink NKG2DLs. **(B)** In situations where opportunistic infection arises, innate immune cells such as macrophages may upregulate surface expression of MHC class I-related chain A (MICA) when activated but mechanisms exist preventing expression of MICA-129met variants, which are retained in cytoplasmic vesicles and degraded. The macrophage expression of MICA-129val may be a source of sMICA due to metalloprotease cleavage from the cell surface. Low-avidity sMICA-129val is then able to compete for binding with NKG2D, reducing the overall net suppressive signal to the NK cell. This may result in the NK cell hyporesponsiveness or tolerance being broken, enabling the NK cell to become activated and help in clearing the opportunistic infection. **(C)** Once the immunological threat has been dealt with, the remaining activated macrophages can be eliminated *via* interaction of NKG2D with cell surface MICA, which stops the production of sMICA-129val. NK cells are then able to return to a suppressed state as sMICB and sULBP1 engage available NKG2D receptors.

The preliminary model, illustrated in Figure 10, allows NK cell suppression due to an abundance of sMICB and sULBP1 ligands within the fetal periphery. Furthermore, sULBP1 is exosomal, multivalent and able to crosslink NKG2D to provide strong signals *via* NKG2D. This is supported by the finding that sULBP1 is strongly correlated with decreased NKG2D expression, NK cell activation, and production of IFN-γ. High levels of sMICB also associated with some of these factors, but its high level of polymorphism may have prevented demonstration of a strong correlation. Macrophages are known to express surface MICA (32–36) and may offer a source of sMICA, detected in around a third of CBP samples, and an innate cellular mechanism of immunity *in utero*. In stark contrast to sMICB and sULBP1, the presence of sMICA resulted in increased activation of NK cells and production of IFN-γ, which was not expected. To understand these results, it is necessary to examine the properties of different NKG2DL and their interaction with NKG2D. MICB and ULBP1 have high affinity for NKG2D and, in the case of sNKG2DLs, deliver a strong suppressive signal to the NK cells. However, MICA can bind NKG2D strongly or weakly depending on the presence of met or val, respectively, at residue 129 (26). In addition, an NK cell-mediated immune response has been described (27, 28, 37), whereby certain mechanisms prevent surface expression of MICA-129met variants, which are retained in cytoplasmic vesicles and degraded. Furthermore, with the exception of MICA-P6, variation in the promoter region of MICA genes does not substantially alter the expression potential as most promoter types resulted in similar transcription levels. The upregulation of MICA-129val by activated fetal macrophages may be a source of sMICA due to metalloprotease cleavage from the cell surface and may also explain the fact that only MICA-129val variants were detectable in CBP and that they were not always present. However, no study has yet demonstrated that macrophages can release sMICA, although little is known of fetal immune system mechanisms. The sudden release of soluble MICA-129val ligands may allow competitive binding with sMICB and sULBP1 for free NKG2D on fetal NK cells, especially as new NK cell progeny emerge. Other unknown mechanisms may also decrease or stop the proteolytic release of fetal sMICB. Although the weaker binding affinity with NKG2D may not be able to outcompete the strong affinity sMICB and sULBP1, it may still be able to bind and prevent their occupation of NKG2D. With NKG2D engagement by sMICA, the net suppressive signal to the cell may be reduced, releasing the NK cell from suppression and enabling it to become activated. The activated fetal NK cells can then proliferate and deal with the infection and once cleared, the release of sMICA-129val ceases. The activated fetal macrophages can then be killed *via* their surface expression of MICA. Soluble MICB and sULBP1 can then dominate occupation of NKG2D on fetal NK cells, which return to their suppressed state due to strong signalling *via* NKG2D.

One problem with this model is that not all individuals express MICA-129val variants as they may be homozygous for MICA-129met or indeed express no MICA at all due to homozygosity of the MICA null allele (38). In this situation, the redundancy of the NKG2DL system may be important to maintain the balance of signals. RAET1G, also known as ULBP5, is an NKG2DL shown to have a similar expression pattern to MICA and can be transcribed as two isomeric forms, RAET1G1 and RAET1G2 (39). RAET1G2 is released as a soluble molecule from the cell and may be used as an alternative to sMICA in this situation. Moreover, RAET1G2 also has a very low affinity for NKG2D, similar to MICA-129val. Although we cannot confirm the presence of soluble RAET1G2 in CBP due to lack of availability of suitable ELISA systems, it may be expressed as we also found sULBP2 and sULBP3 in some CBP samples (10). Therefore, although sMICA may be preferred in immune regulation for unknown reasons, a deficiency of MICA expression with low NKG2D affinity could be compensated by soluble RAET1G2, as it is capable of fulfilling the same function. It is also possible that other sNKG2DLs that were not tested for in this study play a role in modulating NKG2D signalling to release fetal NK cells from suppression. One candidate is sULBP4 as this is the second most polymorphic of the UL-16 binding protein (ULBP) type ligands (3, 5, 40, 41). Binding affinity of ULBP4 for NKG2D is unknown so could be weak and this ligand has recently been shown to have capacity to be expressed as soluble isoforms, either by alternative splicing or proteolytic cleavage (42). We have already typed our cohort of 181 CB samples for ULBP4 allelic polymorphism and are awaiting a suitable and reliable ELISA assay to confirm the presence and level of expression of sULBP4 in CBP.

The preliminary model of fetal NK cell immunity discussed above remains to be confirmed by specific studies to identify the cellular source of sNKG2DLs in cord blood. In addition, the combined effect of different sNKG2DL and their level of expression on the suppression of NK cells need to be investigated in detail as this may represent an important mechanism in fine-tuning the regulation of NK cell immunity in both health and disease.

## Conclusion

Overall, we have found that the main sNKG2DL present in CBP contributing to NK cell suppression is sULBP1. sMICB also reduces NK cell cytotoxicity but it is variable depending on the allelic polymorphism of the promoter or coding region. However, the presence of sMICA in some CBP samples results in increased NK cell function. These findings may relate to mechanisms of fetal–maternal tolerance and *in utero* immunity that are currently poorly understood. Future work should focus on determining the cellular origins of the soluble NKG2DL and also characterise the profile relating to the remaining ULBP4, 5, and 6 ligands and their roles in fetal and maternal immunity.

## Ethics Statement

This study was carried out with the full approval of the local Research Ethics Committee (reference HC71/IU). Peripheral blood was obtained from healthy donors with prior written informed consent. UCB units were obtained from the Anthony Nolan Cord Blood Bank with prior written consent from pregnant mothers and ethical committee approval (Research Ethics Committee reference 10/H0405/27).

## Author Contributions

SC, AS, and JM conceived and designed the study. SC designed and performed experiments, acquired data, performed statistical analysis, interpreted the data, and wrote the manuscript. DH and RD contributed to the administrative, technical, or material support of the study and critically revised the manuscript for important intellectual content. RL-B and HP carried out extensive assay procedures and results analysis. All authors approved the final version of the manuscript.

## Conflict of Interest Statement

Author AS is currently employed by company GlaxoSmithKline. All other authors declare no competing interests.

## Supplementary Material

Figure S1 Gating strategy for determination of natural killer (NK) (CD56^+^CD3^−^) bright and dim cells, NKT cell (CD56^+^CD3^+^), and T cell (CD56^−^CD3^+^) subpopulations and frequency of CD107a-expressing cells. Lymphocyte gating using forward (FSC) and side scatter (SSC) is shown in panel **(A)**. Lymphocyte subpopulations were distinguished using CD56-APC and CD3-PE-Cy7 fluorochromes **(B)**. **(C)** CD107a-expressing cells were gated using SSC against CD107a (FITC) and CD56^+^CD3^−^ NK cells, CD56^+^CD3^+^ NKT cells, and CD56^−^CD3^+^ T cells were gated from this population **(D)**. Double CD56 and CD3-negative cells were designated as “others” for use in Figure 3.

## Abbreviations

CBP, cord blood plasma; MICA, MHC class I-related chain A; MICB, MHC class I-related chain B; MP, maternal plasma; NKG2D, NK group 2, member D; UCB, umbilical cord blood; ULBP, UL-16 binding protein.
