# Corrigendum: Functional Characterisation and Analysis of the Soluble NKG2D Ligand Repertoire Detected in Umbilical Cord Blood Plasma

**DOI:** 10.3389/fimmu.2020.00087

**Published:** 2020-02-04

**Authors:** Steven T. Cox, Robert Danby, Diana Hernandez, Raquel Laza-Briviesca, Hayley Pearson, J. Alejandro Madrigal, Aurore Saudemont

**Affiliations:** ^1^Anthony Nolan Research Institute, Royal Free Hospital, London, United Kingdom; ^2^Cancer Institute, University College London, London, United Kingdom; ^3^Churchill Hospital, Oxford University Hospitals NHS Foundation Trust, Oxford, United Kingdom

**Keywords:** NK cell, pregnancy, tolerance, NKG2D, ligand, luciferase, MICA, ULBP1

**Raquel Laza-Briviesca** and **Hayley Pearson** were not included as authors in the published article. The corrected Author Contributions Statement appears below.

## Author Contributions

SC, AS, and JM conceived and designed the study. SC designed and performed experiments, acquired data, performed statistical analysis, interpreted the data, and wrote the manuscript. DH and RD contributed to the administrative, technical, or material support of the study and critically revised the manuscript for important intellectual content. RL-B and HP carried out extensive assay procedures and results analysis. All authors approved the final version of the manuscript.

The authors apologize for this error and state that this does not change the scientific conclusions of the article in any way. The original article has been updated.

