# Supplementary figures and images for: Functional Characterisation and Analysis of the Soluble NKG2D Ligand Repertoire Detected in Umbilical Cord Blood Plasma
Source: Front Immunol. 2018 Jun 15;9:1282. doi: 10.3389/fimmu.2018.01282 (PMC6013648; doi:10.3389/fimmu.2018.01282)

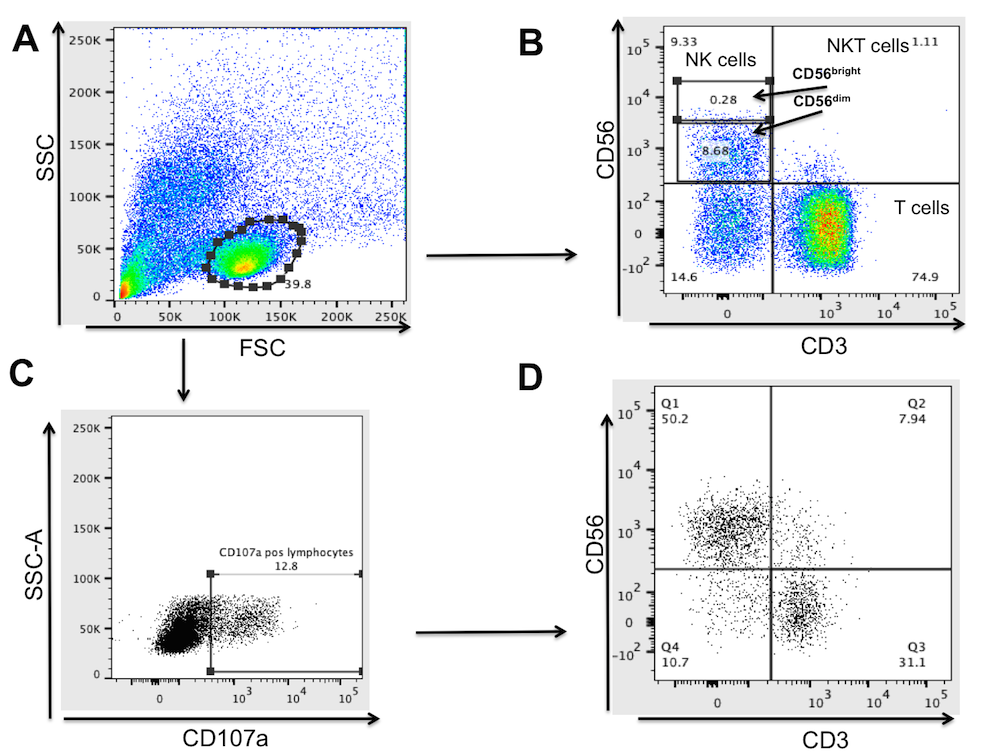

Supplement: Figure S1 — Gating strategy for determination of natural killer (NK) (CD56+CD3−) bright and dim cells, NKT cell (CD56+CD3+), and T cell (CD56−CD3+) subpopulations and frequency of CD107a-expressing cells. Lymphocyte gating using forward (FSC) and side scatter (SSC) is shown in panel (A). Lymphocyte subpopulations were distinguished using CD56-APC and CD3-PE-Cy7 fluorochromes (B). (C) CD107a-expressing cells were gated using SSC against CD107a (FITC) and CD56+CD3− NK cells, CD56+CD3+ NKT cells, and CD56−CD3+ T cells were gated from this population (D). Double CD56 and CD3-negative cells were designated as “others” for use in Figure 3. [file Image_1.tiff]
